# Effects of Different Moments of Inertia on Neuromuscular Performance in Elite Female Soccer Players During Hip Extension Exercise to Prevent Hamstring Asymmetries and Injuries: A Cross-Sectional Study

**DOI:** 10.3390/sports13070212

**Published:** 2025-06-28

**Authors:** Jordi Pumarola, Alesander Badiola-Zabala, Mònica Solana-Tramunt

**Affiliations:** 1 Global Performance, 08022 Barcelona, Spain; jordipp6@blanquerna.url.edu; 2 Facultat de Psicologia, Ciències de l’Educació i l’Esport Blanquerna, Universitat Ramon Llull, 08022 Barcelona, Spain; 3 Facultat de Ciències de la Salut Blanquerna, Universitat Ramon Llull, 08022 Barcelona, Spain

**Keywords:** cone-shaped prevention exercise, iso-inertial devices, inertia load, power output, acceleration output, speed output, hamstrings asymmetry

## Abstract

Background: High-intensity actions like accelerations and decelerations, often performed unilaterally, are crucial in elite female football but increase the risk of interlimb asymmetries and injury. Flywheel resistance training enhances eccentric strength, yet limited research has assessed how different inertial loads affect mechanical outputs in unilateral exercises. Purpose: This study investigated how two inertial loads (0.107 kg·m^2^ and 0.133 kg·m^2^) influence power, acceleration, speed, and asymmetry during unilateral hip extensions in elite female footballers. Methods: Eighteen professional players (27 ± 4 years, 59.9 ± 6.5 kg, 168.2 ± 6.3 cm, BMI 21.2 ± 1.8) completed unilateral hip extensions on a conical flywheel under both inertia conditions. A rotary encoder measured peak/average power, acceleration, speed, and eccentric-to-concentric (E:C) ratios. Bilateral asymmetries between dominant (DL) and non-dominant (NDL) limbs were assessed. Paired t-tests and Cohen’s *d* were used for analysis. Results: Higher inertia reduced peak and mean acceleration and speed (*p* < 0.001, *d* > 0.8). Eccentric peak power significantly increased in the NDL (*p* < 0.001, *d* = 3.952). E:C ratios remained stable. Conclusions: Greater inertial loads reduce movement velocity but increase eccentric output in the NDL, offering potential strategies to manage neuromuscular asymmetries in elite female football players.

## 1. Introduction

High-speed running and high-intensity actions are essential components of physical performance in elite international female football [1]. Within these demanding contexts, the ability to effectively execute accelerations and decelerations plays an essential role, significantly contributing to the total distance covered at high intensities, including sprints, during matches [2]. Since many of these actions are performed unilaterally, the development of interlimb asymmetries is anticipated, as evidenced by previous research which demonstrated that interlimb asymmetries are associated with a decrease in athletic performance [3,4,5]. To meet these physical demands, the implementation of well-designed strength and conditioning programs is crucial, as they not only enhance long-term physical capacities but also help mitigate the risk of injuries [6].

One promising strategy for optimizing athletic performance is the use of specialized training equipment that enhances power output during sprinting, focusing particularity on the acceleration and deceleration phases with an emphasis on eccentric (E) loading [7]. Traditional resistance training methods, such as the use of free weights, often do not sufficiently stimulate the eccentric (E) phase, missing opportunities to maximize the unique adaptations linked with E contractions [8]. To address this gap, flywheel training devices—especially cone-shaped versions—have been incorporated into training programs to target improvements during the E phase, thereby enhancing overall performance [9,10].

The mechanics of flywheel resistance training rely on the kinetic energy produced during the concentric (C) phase, requiring a comparable impulse to decelerate the rotational movement of the flywheel [11]. To maximize training efficiency, athletes are encouraged to exert maximal force during the C acceleration phase, followed by an effective braking action during the E deceleration phase to generate force enhancement in the subsequent acceleration phase [11]. Achieving E overload, which is defined as having an eccentric-to-concentric ratio (E:C) greater than one, is a key goal in flywheel training, as it leads to superior neuromuscular adaptations compared to C-dominant training [12]. Selecting an appropriate internal load is critical to achieving this overload and thus maximizing training outcomes [6].

Recent studies have sought to determine the impact of varying moments of inertia on crucial training metrics such as peak power, to improve training results and optimize load management [13,14,15]. For example, research has shown that unilateral knee extensions performed with higher moments of inertia (0.075, 0.1 kg·m^2^) produce greater peak power in both C and E phases compared to lower inertial loads (0.0125, 0.025, 0.0375 kg·m^2^) [16]. On the other hand, exercises like squats and deadlifts tend to generate higher peak power at lower moments of inertia, indicating that the responses to different inertial loads may be exercise-specific [13,15,17].

The distinct responses of different exercises to changes in moments of inertia have sparked interest in understanding whether similar outcomes are observed for other lower-body exercises, such as leg curls and hip extensions, which target different parts of the hamstring force–length curve [18,19].

For instance, Keijzer et al. [20] demonstrated that, in unilateral leg curls, a wide range of moments of inertia could elicit high E demands, whereas hip extension required greater inertia to achieve E overload. Importantly, these investigations used flywheel devices with inertia values ranging from 0.029 to 0.089 kg·m^2^, highlighting the need for further explorations into how these parameters influence performance outcomes [6].

Despite the increasing popularity of flywheel training for enhancing E strength, there is still a lack of comprehensive guidelines for its application, particularly within the context of soccer [6,21]. Peak power remains the primary variable used to quantify and monitor training loads in flywheel-based protocols, and the E:C ratio has proven to be a reliable measure for assessing eccentric overload when peak power values are used as an absolute metric [22]. However, additional research is needed to establish comprehensive guidelines for optimizing flywheel training across different muscle groups, thereby enhancing the efficacy of training management and prescription practices. Therefore, the primary objective of this study was to examine the effects of varying moments of inertia during the hamstring extension exercise performed with a cone-shaped flywheel device. Specifically, the study aims to evaluate how different inertia settings influence peak and average mechanical outputs and the E:C ratio. The secondary objective was to assess the effect of inertia moments on interlimb asymmetries. We hypothesized that lower moments of inertia will yield higher peak and average mechanical values. As for neuromuscular asymmetries, they will be independent of the external resistance.

## 2. Materials and Methods

### 2.1. Study Design

A randomized, repeated-measures cross-sectional design was employed. The study was conducted in accordance with the STROBE reporting guidelines [23].

### 2.2. Study Population

A total of eighteen female professional football players from the elite Spanish division were enrolled in this study, with participants specifying their age (27 ± 4 years), height (168.2 ± 6.3 cm), and weight (59.9 ± 6.5 kg) (Table 1). The primary inclusion criteria required a minimum of 6 months of engagement in weekly resistance training programs focusing on lower body muscles, consistent with prior research emphasizing eccentric overload [14]. Consequently, regular participation in five pitch training sessions per week was mandatory. The main exclusion criteria comprised instances of lower body muscle or neuronal injury within the preceding 6 months and the use of drugs or any medication that could vary the results during the testing session. Participants received prior exposure to eccentric overload (EOL) exercises and test procedures as integral components of their football training and weekly testing routine (Table 2). All the participants were apprised of the potential risks and benefits of the study procedures and provided written informed consent. Ethical approval for the present study was obtained from the research ethics committee of the University of Blanquerna-Ramon Llull (Barcelona, Spain). All the procedures adhered to the principles outlined in the Declaration of Helsinki for studies involving human subjects [24].

### 2.3. Data Collection

All data collection took place on the same day during the first part of the season in October 2024. Measurements were conducted at the team’s regular training facilities. Prior to the evaluation day, the researchers coordinated with the head coach to schedule the timing of each measurement, ensuring minimal disruption to the field training and enhancing the ecological validity of the study. Data collection from the conical pulley was achieved using a high-resolution rotary encoder (NexSo, Neuroexcellence, Braga, Portugal) connected via Bluetooth to the data software app (NexSo Software version 1.5.9, Neuroexcellence, Braga, Portugal). The encoder recorded data from each repetition in both the concentric and eccentric phases. The data analysis focused on variables such as concentric peak power, eccentric peak power, eccentric–concentric ratio (E:C ratio), and the mean power of the concentric and eccentric phases. The same analysis was conducted for acceleration and speed for the peak and average outputs. The E:C ratio was calculated using the average from the three fastest concentric speed repetitions of the set [14].

### 2.4. Testing Procedures

**Figure 1:**
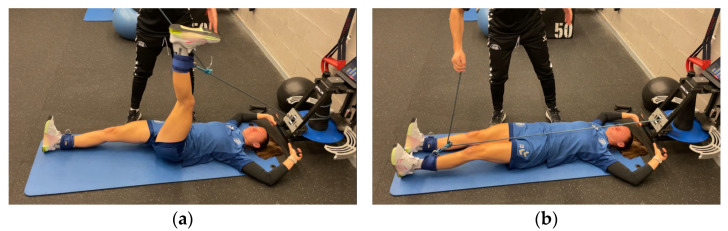
(**a**) Initial position for the hip extension exercise using flywheel cone-shape device (90° hip flexion and knee semi-flexion). (**b**) Final position for the hip extension exercise using flywheel cone-shape device (hip and knee at 0°).

**Figure 2:**
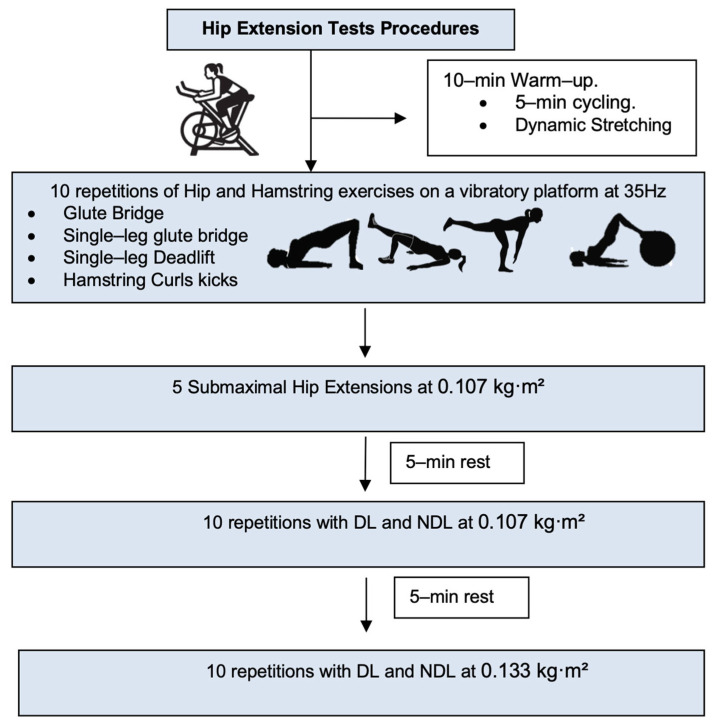
Testing procedures. DL = dominant leg. NDL = non–dominant leg.

The participants performed hip extensions using a flywheel cone-shaped device (Cyclon Cone S4, GPServices, Barcelona, Spain; Figure 1a,b) in two sets with distinct inertial loads (0.107 and 0.133 kg·m^2^). Prior to testing, they completed a standardized 10 min warm-up: 5 min of stationary cycling, followed by dynamic stretching and mobilization of the hip and knee joints. Subsequently, four exercises were performed on a 35 Hz vibratory platform (Power Move, GPServices): Glute Bridge, Single-Leg Glute Bridge, Single-Leg Deadlift, and Hamstring Curl Kicks on a Swiss ball (10 reps each). A submaximal familiarization set of five bilateral hip extensions at the lowest load (0.107 kg·m^2^) was then conducted. Testing began with the dominant limb (DL), followed by the non-dominant limb (NDL), with a 5 min rest between sets [14]. Each set included 2–3 initial accelerative repetitions followed by up to 10 maximal-effort reps (Figure 2). During the concentric phase, the participants maximized voluntary effort to accelerate the disc and minimized transition time to enhance the eccentric overload (EOL). The participants were supine, head 10 cm from the device, gripping lateral handles. The lumbar spine remained in contact with the floor throughout. The working leg performed hip flexion with the knee semi-flexed (~15°). The dominant leg was determined by functional capability.

### 2.5. Statistical Analysis

All statistical analyses were conducted using JASP software for Apple (version 0.18.3, Amsterdam, Netherlands). Data normality was assessed using the Shapiro–Wilk test, and variables violating normality assumptions (*p* < 0.05) were further analyzed using Wilcoxon signed-rank tests as a non-parametric alternative. For normally distributed variables, paired samples t-tests were employed to compare the effects of different moments of inertia (0.107 kg·m^2^ and 0.133 kg·m^2^) on concentric and eccentric peak and average power, acceleration, and speed, as well as eccentric-to-concentric (E:C) ratio and neuromuscular asymmetry percentage (%ASI). Effect sizes were calculated using Cohen’s d, categorized as follows, as Hopkins et al. [25] suggest: <0.20 (trivial), 0.2–0.59 (small), 0.6–1.19 (moderate), 1.2–2.0 (large), and >2.0 (very large).

## 3. Results

### 3.1. Peak Mechanical Outputs Dominant and Non-Dominant Limb Comparisons with Different Moments of Inertia (0.107 kg·m^2^ and 0.133 kg·m^2^)

Mean and standard deviation from all peak mechanical outputs (power, angular acceleration and angular speed) with two moments of inertia (107 kg·m^2^ and 0.133 kg·m^2^) are presented in Table 3.

**Figure 3:**
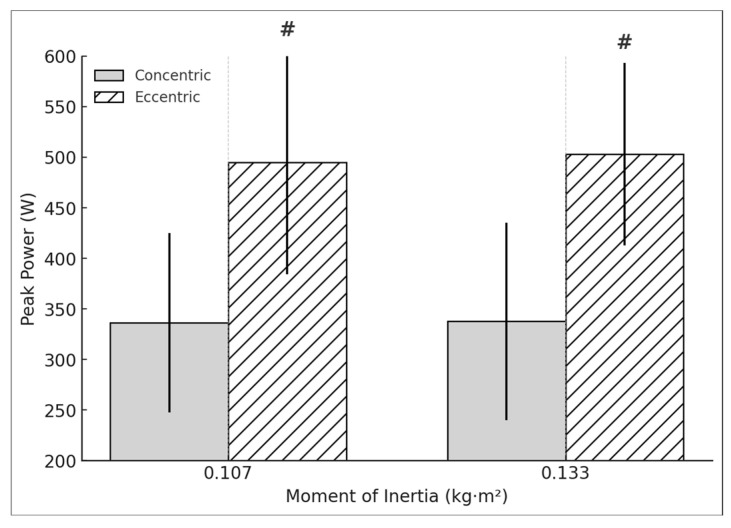
NDL concentric and eccentric peak power during the hip extension exercise using a flywheel cone-shape device. # Statistically significant (*p* < 0.05) difference between moments of inertia for the concentric phase.

The DL showed no significant differences in concentric peak power between resistances (t = −2.064, *p* = 0.054, d = −0.473). Likewise, no significant differences were observed in eccentric peak power (t = −0.461, *p* = 0.65, d = −0.106). The E:C ratio for peak power did not reach statistical significance (t = 1.442, *p* = 0.166, d = 0.331). The NDL showed no significant differences in concentric peak power between resistances (t = −0.07, *p* = 0.945, d = −0.016). However, a very large significant relation was found in eccentric peak power (t = 17.674, *p* < 0.001, d = 3.952) (Figure 3). The E:C ratio for peak power did not show significant changes (t = −0.665, *p* = 0.514, d = −0.149). No significant differences were found in the asymmetry between phases and loads (t = −1.257, *p* = 0.225, d = −0.288) for the concentric phase and eccentric phase (t = −0.17, *p* = 0.867, d = −0.039).

**Figure 4:**
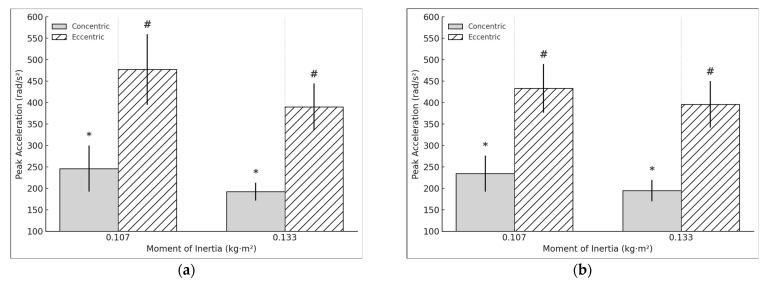
(**a**) DL concentric and eccentric peak acceleration during the hip extension exercise using a flywheel cone-shape device. * # Statistically significant (*p* < 0.05) difference between moments of inertia for the concentric phase. (**b**) NDL concentric and eccentric peak acceleration during the hip extension exercise using a flywheel cone-shape device. * # Statistically significant (*p* < 0.05) difference between moments of inertia for the concentric phase.

For the acceleration, a moderate significant difference in concentric peak acceleration was observed in the DL when performing the exercise at 0.133 kg·m^2^, compared to 0.107 kg·m^2^ (t = 4.098, *p* < 0.001, d = 0.94). Similarly, a moderate significant relation was found in eccentric peak acceleration (t = 4.967, *p* < 0.001, d = 1.14) (Figure 4a). However, the E:C ratio for acceleration did not show significant changes (t = −1.42, *p* = 0.173, d = −0.326). In the NDL, moderate significant differences were observed in both concentric peak acceleration (t= 4.026, *p* < 0.001, d =0.9) and eccentric peak acceleration (t = 3.862, *p* = 0.001, d = 0.864) (Figure 4b). Conversely, the E:C ratio for acceleration showed a small negative significant difference (t = −2.218, *p* = 0.039, d = −0.496). Regarding asymmetry in acceleration, no significant differences were found in the concentric phase (t = 0.936, *p* = 0.362, d = 0.215). However, a small significant positive relation in eccentric acceleration asymmetry was observed (t = 2.325, *p* = 0.032, d = 0.533).

**Figure 5:**
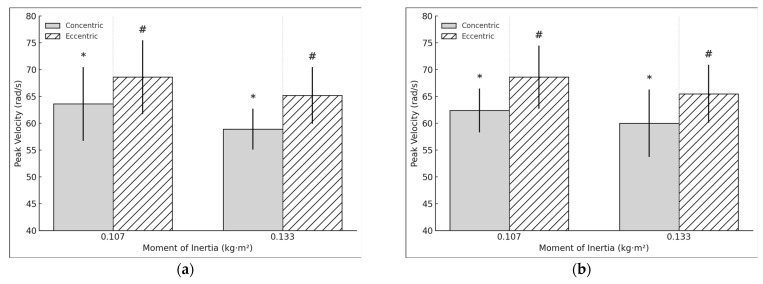
(**a**). DL concentric and eccentric peak speed during the hip extension exercise using a flywheel cone-shape device. * # Statistically significant (*p* < 0.05) difference between moments of inertia for the concentric phase. (**b**) NDL concentric and eccentric peak speed during the hip extension exercise using a flywheel cone-shape device.

For the speed, a moderate significant relation in concentric peak speed was observed in the DL when performing the exercise at 0.133 kg·m^2^, compared to 0.107 kg·m^2^ (t = 3.493, *p* = 0.003, d = 0.81) (Figure 5a). Similarly, a moderate significant relation was found in eccentric peak speed (t = 3.73, *p* =0.002, d = 0.856) (Figure 5b). However, the E:C ratio for speed did not show significant changes (t = −1.694, *p* = 0.108, d = −0.389). In the NDL, moderate significant relations were observed in both concentric peak speed (t = 2.235, *p* = 0.0038, d = 0.513) and eccentric peak speed (t = 3548, *p* = 0.002, d = 0.793). The E:C ratio for speed did not show significant differences (t = 0.635, *p* = 0.533, d = 0.142). Regarding asymmetry in speed, no significant differences were found in the concentric phase (t = 1.47, *p* = 0.159, d = 0.337) or the eccentric phase (t = 0.317, *p* = 0.755, d = 0.073).

### 3.2. Mean Mechanical Outputs Dominant and Non-Dominant Limb Comparisons with Different Moments of Inertia (0.107 kg·m^2^ and 0.133 kg·m^2^)

Mean and standard deviation from all mean mechanical outputs (power, angular acceleration, and angular speed) with two moments of inertia (107 kg·m^2^ and 0.133 kg·m^2^) are presented in Table 4.

No significant differences were found in concentric (t = 0.672, *p* = 0.51, d = 0.154) or in eccentric average power (t = −0.556, *p* = 0.585, d = −0.128) for the DL, nor in the E:C ratio (t = −1.253, *p* = 0.226, d = −0.287). In the NDL, concentric (t = −1.237, *p* = 0.231, d = −0.277) and eccentric average power (t = −0.774, *p* = 0.448, d = −0.173) also showed no significant changes, with the E:C ratio remaining stable (t = 0.389, *p* = 0.701, d = 0.087). Similarly, asymmetry in average power was unaffected in both the concentric (t = 1.326, *p* = 0.202, d = 0.304) and eccentric phases (t = 0.004, *p* = 0.965, d = 0.01).

**Figure 6:**
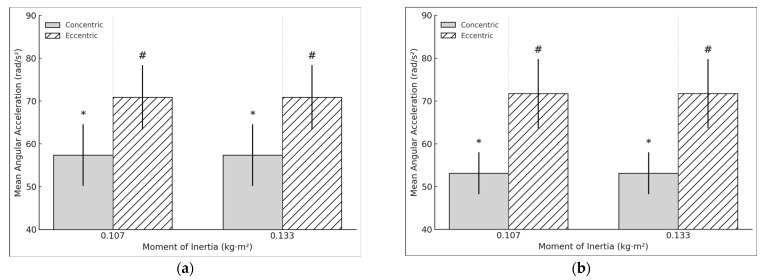
(**a**) DL concentric and eccentric mean acceleration during the hip extension exercise using a flywheel cone-shape device. * # Statistically significant (*p* < 0.05) difference between moments of inertia for the concentric phase. (**b**) NDL limb concentric and eccentric mean acceleration during the hip extension exercise using a flywheel cone-shape device.

Concentric (t = 7.238, *p* < 0.001, d = 1.661) and eccentric average acceleration (t = 5.43, *p* < 0.001, d = 1.246) had a large significant relation in the DL at 0.133 kg·m^2^ (Figure 6a). However, the E:C ratio showed a very large negative significant relation (t = −18.344, *p* < 0.001, d = −4.208). In the NDL, a large significant relation was also found in concentric (t = 6.44, *p* < 0.001, d = 1.44) and eccentric acceleration (t = 8.6, *p* < 0.001, d = 1.923), while the E:C ratio remained unchanged (t = −1.787, *p* = 0.091, d = −0.41) (Figure 6b). No significant differences were found in asymmetry during the concentric (t = 1.864, *p* = 0.079, d = 0.428) or eccentric phase (t = 0.044, *p* = 0.965, d = 0.01).

**Figure 7:**
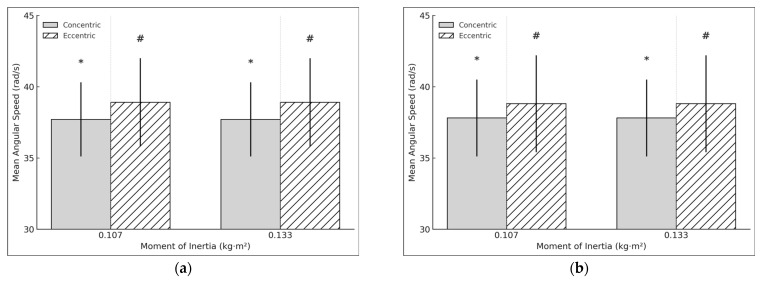
(**a**) DL concentric and eccentric mean velocity during the hip extension exercise using a flywheel cone-shape device. * # Statistically significant (*p* < 0.05) difference between moments of inertia for the concentric phase. (**b**) NDL concentric and eccentric mean velocity during the hip extension exercise using a flywheel cone-shape device.

Concentric (t = 9.861, *p* < 0.001, d = 2.262) and eccentric average speed (t = 4.464, *p* < 0.001, d = 1.024) had a very large and large significant relation in the DL at 0.133 kg·m^2^, while the E:C ratio remained non-significant (t = −1886, *p* < 0.075, d = −0.433) (Figure 7a). In the NDL, concentric (t = 9.813, *p* < 0.001, d = 2.194) and eccentric average speed (t = 5.29, *p* < 0.001, d = 1.183) also had a large relation, though the E:C ratio showed a large significant relation (t = 5.684, *p* < 0.001, d = 1.271) (Figure 7b). No significant differences were found in asymmetry for either the concentric (t = 0.386, *p* = 0.704, d = 0.089) or eccentric phase (t = 0.349, *p* = 0.731, d = 0.08).

## 4. Discussion

This study explored how different inertial loads (0.107 kg·m^2^ vs. 0.133 kg·m^2^) influence mechanical outputs during unilateral hip extension in elite female football players, with particular focus on concentric/eccentric demands and interlimb asymmetry. While the results demonstrated clear load-dependent changes in mechanical performance, the discussion below highlights the physiological and practical implications of these outcomes, avoiding unnecessary repetition of statistical values.

Our findings confirm the well-established principle that increased inertial loads amplify eccentric demands during flywheel exercises, particularly evident in the non-dominant limb. This aligns with the prior literature [17,26], reinforcing the concept of eccentric overload as a key benefit of flywheel training. Interestingly, concentric outputs (power, speed, acceleration) were less sensitive to changes in inertia, especially in peak power, suggesting a ceiling effect or task-specific adaptation that may limit the utility of concentric variables in load progression.

Acceleration and speed increased under higher loads across both limbs, reflecting greater neuromuscular demand and a likely increase in motor unit recruitment [27]. However, the eccentric-to-concentric (E:C) ratios for power, speed, and acceleration remained relatively stable, questioning their sensitivity as indicators of training intensity—an observation previously noted by Maroto-Izquierdo et al. [26]. Consequently, absolute eccentric values may offer more actionable insight for load prescription than E:C metrics.

The asymmetry analysis revealed more pronounced differences during eccentric rather than concentric actions, supporting the hypothesis that unilateral eccentric control may expose neuromuscular imbalances. This is particularly relevant for injury prevention and return-to-play strategies, as asymmetric eccentric performance may reflect underlying deficits in coordination, strength, or motor control [15,26].

### 4.1. Practical Implications

From a performance perspective, these results suggest that manipulating inertial loads allows targeted control of mechanical stimuli during training. For strength and conditioning professionals, heavier inertial settings may serve to prioritize eccentric strength development while moderating speed and acceleration—useful in rehabilitation or off-season contexts where joint load management is essential. Conversely, lower loads may be used to develop speed and movement efficiency without excessive muscular strain.

Importantly, given the minimal changes in E:C ratios, practitioners should rely on direct measurements (e.g., eccentric power or acceleration) rather than derived metrics when evaluating or adjusting training stimuli.

### 4.2. Limitations and Future Directions

Several limitations must be acknowledged. First, the sample size, although homogeneous, may limit generalizability. Second, this study focused solely on hip extension—the findings may not extrapolate to compound or multi-joint exercises. Third, baseline strength levels were not normalized, which may have influenced individual responses. Moreover, eccentric effort was not standardized across participants, and no electromyographic (EMG) data were collected to verify neuromuscular activation patterns.

Future studies should incorporate EMG, motion capture, and effort quantification tools to better understand interlimb differences and their neuromechanical underpinnings. Longitudinal designs could assess how chronic exposure to different inertias modulates asymmetry and performance. Additionally, implementing individualized load prescriptions based on strength assessments may enhance internal validity.

## 5. Conclusions

This study demonstrates that increasing the moment of inertia during unilateral flywheel hip extension exercise significantly decreased peak and mean acceleration and velocity in both concentric and eccentric phases, for both the DL and NDL. Additionally, eccentric peak power significantly increased only in the NDL, while concentric peak power and E:C ratio remained stable across conditions. These findings suggest that higher inertial loads may be a practical tool to control movement velocity and neuromuscular demands during training sessions, allowing strength and conditioning professionals to better align exercise intensity with specific performance goals. Although bilateral symmetry was largely preserved, small but significant asymmetries appeared in eccentric accelerations and speed, indicating the importance of individualized monitoring. Finally, absolute eccentric outputs may provide more reliable markers for training regulation than E:C ratios in the context of unilateral flywheel training, for elite female football players’ neuromuscular performance, and highlight areas for future research in flywheel exercises aimed at performance enhancement and injury prevention.

## Figures and Tables

**Table 1:** Demographic characteristics of the participants.

Variables	Mean ± SD
Age (years)	27 ± 4
Weight (kg)	59.9 ± 6.5
Height (cm)	168.2 ± 6.3
IMC	21.2 ± 1.8

**Table 2:** Inclusion criteria.

Inclusion Criteria
Minimum of 6 months of engagement in weekly resistance training programs focusing on lower body muscles. Regular participation in five pitch training sessions per week. Free of lower body muscle or neuronal injury within the preceding 6 months. Free of drugs or any medication that could vary the results during the testing session.

**Table 3:** Flywheel cone-shape device unilateral hip extension mean ± SD from peak mechanical outputs.

	Load (kg·m^2^)			
Variable	0.107		0.133	
	Concentric	Eccentric	Concentric	Eccentric
	Mean (SD)	Mean (SD)	Mean (SD)	Mean (SD)
DL Power	316.8	503	350.7	511
(W)	(49.9)	(114.3)	(111.9)	(91.6)
NDL Power	336.2	495.1	337.7	503
(W)	(88.7)	(110.9)	(97.7)	(90.5)
DL Angular acceleration	246.1	477.2	192.3	390.2
(rad/s^2^)	(53.9)	(82.5)	(20.7)	(42.5)
NDL Angular acceleration	234.3	443.1	194.9	395.8
(rad/s^2^)	(41.9)	(57.1)	(25.1)	(54.2)
DL Angular Speed	63.6	68.6	58.9	65.2
(rad/s)	(6.9)	(6.9)	(3.8)	(5.3)
NDL Angular speed	62.4	68.6	60	65.5
(rad/s)	(4.1)	(5.9)	(6.3)	(5.4)

Results are expressed by mean (standard deviation: SD). Dominant limb (DL); non–dominant limb (NDL).

**Table 4:** Flywheel cone-shape device unilateral hip extension mean ± SD from mean mechanical outputs.

	Load (kg·m^2^)			
Variable	0.107		0.133	
	Concentric	Eccentri	Concentric	Eccentric
	Mean (SD)	Mean (SD)c	Mean (SD)	Mean (SD)
DL Power	155.8	173.8	153.9	173.8
(W)	(24.2)	(27.2)	(22.1)	(27.3)
NDL Power	149.7	172.1	149.7	172.1
(W)	(23.9)	(29.7)	(23.9)	(29.7)
DL Angular acceleration	57.4	70.9	57.4	70.9
(rad/s^2^)	(7.2)	(7.5)	(7.2)	(7.5)
NDL Angular acceleration	53.1	71.7	53.1	71.7
(rad/s^2^)	(4.9)	(8.1)	(4.9)	(8.1)
DL Angular Speed	37.7	38.9	37.7	38.9
(rad/s)	(2.6)	(3.1)	(2.6)	(3.1)
NDL Angular speed	37.8	38.8	37.8	38.8
(rad/s)	(2.7)	(3.4)	(2.7)	(3.4)

Results are expressed by mean (standard deviation: SD). Dominant limb (DL); non–dominant limb (NDL).

## Data Availability

The raw data supporting the results and conclusions of this article will be made available by the authors, without undue reservation.
